# Influence of centrifugation conditions on the results of 77 routine clinical chemistry analytes using standard vacuum blood collection tubes and the new BD-Barricor tubes

**DOI:** 10.11613/BM.2018.010704

**Published:** 2017-11-24

**Authors:** Janne Cadamuro, Cornelia Mrazek, Alexander B. Leichtle, Ulrike Kipman, Thomas K. Felder, Helmut Wiedemann, Hannes Oberkofler, Georg M. Fiedler, Elisabeth Haschke-Becher

**Affiliations:** 1 Department of Laboratory Medicine, Paracelsus Medical University, Salzburg, Austria; 2 University Institute of Clinical Chemistry, Inselspital, Bern University Hospital, University of Bern, Switzerland; 3 UT SPSS Statistics, Hallein, Austria

**Keywords:** pre-analytics, centrifugation, diagnostic tests, routine

## Abstract

**Introduction:**

Although centrifugation is performed in almost every blood sample, recommendations on duration and g-force are heterogeneous and mostly based on expert opinions. In order to unify this step in a fully automated laboratory, we aimed to evaluate different centrifugation settings and their influence on the results of routine clinical chemistry analytes.

**Materials and methods:**

We collected blood from 41 healthy volunteers into BD Vacutainer PST II-heparin-gel- (LiHepGel), BD Vacutainer SST II-serum-, and BD Vacutainer Barricor heparin-tubes with a mechanical separator (LiHepBar). Tubes were centrifuged at 2000xg for 10 minutes and 3000xg for 7 and 5 minutes, respectively. Subsequently 60 and 21 clinical chemistry analytes were measured in plasma and serum samples, respectively, using a Roche COBAS instrument.

**Results:**

High sensitive Troponin T, pregnancy-associated plasma protein A, ß human chorionic gonadotropin and rheumatoid factor had to be excluded from statistical evaluation as many of the respective results were below the measuring range. Except of free haemoglobin (fHb) measurements, no analyte result was altered by the use of shorter centrifugation times at higher g-forces. Comparing LiHepBar to LiHepGel tubes at different centrifugation setting, we found higher lactate-dehydrogenase (LD) (P = 0.003 to < 0.001) and lower bicarbonate values (P = 0.049 to 0.008) in the latter.

**Conclusions:**

Serum and heparin samples may be centrifuged at higher speed (3000xg) for a shorter amount of time (5 minutes) without alteration of the analytes tested in this study. When using LiHepBar tubes for blood collection, a separate LD reference value might be needed.

## Introduction

Apart from sample collection and transportation, sample preparation by centrifugation is one of the most critical steps in the pre-analytical phase. In order to obtain the best sample material quality for laboratory analysis centrifugation time, speed and temperature are crucial. Prolonged centrifugation at high speed might lead to haemolysis or structural damage to the measurand whereas brief low speed centrifugations may lead to insufficient separation of plasma or serum from cellular blood components (*1*). As a consequence, laboratory analyses might be altered due to chromatic interferences, loss of analytes being metabolized or consumed and interference by residual cellular components such as platelets, leucocytes or their components (*2*-*5*). In addition to these quality issues of sample preparation, many if not all laboratories have to keep turnaround times (TATs) in the total testing process as short as possible in order to rapidly diagnose the patient. As the duration for the analytical process usually is invariably determined by the respective analytical device, reducing the TAT within the laboratory may be achieved by optimizing the centrifugation conditions. One obstacle in such optimization attempts is the fact that these conditions depend on the type of sample or even on the type of separator. For example, coagulation tubes should usually be centrifuged for a longer period of time than samples for clinical chemistry analyses to retrieve platelet poor or even platelet “free” plasma (*6*). In current recommendations on sample centrifugation, there is a big variety in terms of centrifugation time, speed and temperature, ranging from 10 to 20 minutes and 1500 to 3000xg at 15 to 25°C, respectively (*6*-*13*). These parameters also change depending on the gel composition used in the specific country with g-forces as low as 1000xg (*14*). The manufacturer of the newly introduced BD-Barricor tube, which uses a mechanical separator instead of a gel barrier, claims that these tubes may be centrifuged at higher speed and shorter time, recommending centrifugation for 5 minutes at 3000xg or only 3 minutes at 4000xg.

Often it is referred to the centrifugation recommendation of blood collection tube manufacturers (*15*). However, picturing the number of possible laboratory parameters on all of the possible analytical platforms, it seems unlikely that providers of blood collection tubes are able to validate every single one of these combinations. Even if such a validation would be available, tube manufacturers would have to find a way of validating also newly introduced parameters measured in plasma or serum, collected into one of their tubes. Therefore tube vendors most probably recommend to centrifuge for a longer duration than necessary to prevent incomplete plasma/serum separation and to cover most if not all possible analytical combinations (*11*-*13*). We therefore believe that manufacturers of analytical devices or assays should validate sample preparation settings for their products, taking tube characteristics in terms of material or resistance to temperature and g-forces into account, instead of relying on recommendations of tube manufacturers. However, these validation data are often lacking.

In our hospital Vacutainer, LH PST^TM^ II lithium heparin and Vacutainer, SST^TM^ II Advance serum tubes as well as the Vacutainer Barricor^TM^ tubes (all tubes from Becton Dickinson, Franklin Lakes, NJ/USA), which are used in our emergency department, are centrifuged for 10 minutes at 2000xg. As we aim to implement fully automated pre-analytical and analytical processes, we were seeking to achieve a unified and standardized sample centrifugation with the shortest time possible for most if not all sample types without significantly altering plasma/serum quality. In the field of haemostasis, respective evaluations of shortened centrifugation time at higher g-forces are available in recent literature (*3*, *16*-*18*). For clinical chemistry parameters however, these data are scarce (*19*).

In this study we therefore aimed to evaluate a standardized sample preparation protocol for clinical chemistry analyses by comparing different centrifugation conditions in heparin-gel-, serum- and Barricor tubes.

## Materials and methods

This study was conducted at the Department of Laboratory Medicine and Microbiology of the University Hospital Salzburg between August and November 2016.

**Figure 1:**
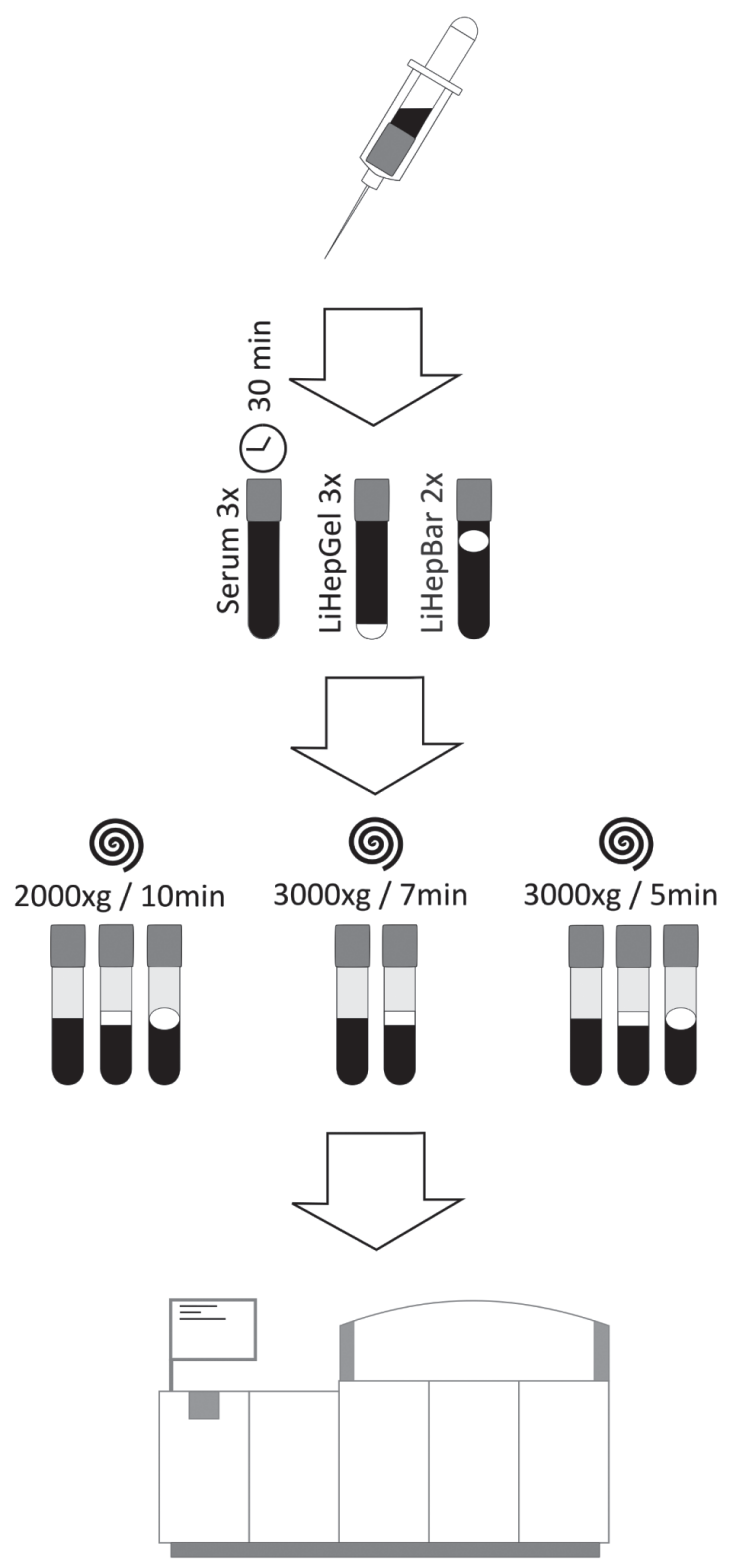
Sample collection and preparation workflow. LiHepGel – vacuum lithium heparin gel tubes. LiHepBar – vacuum lithium heparin Barricor tubes.

After approval by the local ethics committee (Protocol number 415-E/2028/9-2016) and a written informed consent of every participant, three Lithium-Heparin gel tubes (LiHepGel) (BD Vacutainer, LH PST^TM^ II, 16x100mm, 8ml REF:367378), three serum gel tubes (BD Vacutainer, SST^TM^ II Advance, 16x100mm, 8ml REF: 367953) and two Lithium Heparin tubes using a mechanical separator (LiHepBar) (BD Vacutainer Barricor LH Plasma, 13x100mm, 5ml REF: 365039) were drawn from 41 healthy volunteers (17 males and 24 females with a median (range) age of 42 (20-74) years) according to current recommendations (*20*). All blood collections were performed using a 21G winged safety blood collection set (GBO Vacuette safety blood collection set, 19cm, REF 450081; Greiner BioOne, Kremsmünster, Austria). The order of sample tube collection was randomized in every participant and the tourniquet was removed while the first tube was filled. All samples were mixed according to the manufacturer’s instructions. LiHepGel and LiHepBar tubes were processed immediately after sample collection. Serum samples were stored at room temperature in an upright position for 30 minutes to allow the sample to clot appropriately before further processing (*21*). Always one of the respective LiHepGel and serum tubes were centrifuged at 2000xg for 10 minutes, as well as at 3000xg for 7 minutes and at 3000xg for 5 minutes. The two LiHepBar tubes were centrifuged at 2000xg for 10 minutes and at 3000xg for 5 minutes (Figure 1). There was no LiHepBar tube being centrifuged at 3000xg for 7 minutes as recommendations emphasize an even shorter time of 5 minutes at this g-force. High throughput centrifuges as used in our laboratory mostly reach a maximum of about 3000xg, therefore no higher g-forces were evaluated. All centrifugations were performed on Hettich centrifuges (Rotanta 460R and Rotixa 500RS, Hettich Lab Technology, Tuttlingen, Germany) using a swing-out rotor at a temperature of 22°C. Sixty analytes were measured in heparin plasma and another 21 analytes were measured in serum. Analyses for all of the 81 parameters, measurable on the Roche COBAS 8000 device (Roche Diagnostics, Rotkreuz, Switzerland) were performed in duplicate, immediately after centrifugation (Table 1).

**Table 1:** Comparison of laboratory values measured in plasma for different centrifugation conditions and tube type

**Parameter, unit**	N*	**CV (%)^†^**	**Centrifugation conditions per tube type**					**P**
**2000xg/10min**	**2000xg/10min**	**3000xg/7min**	**3000xg/5min**	**3000xg/5min**
**LiHepGel**	**LiHepBar**	**LiHepGel**	**LiHepGel**	**LiHepBar**
Chloride, mmol/L	41	1.6	98 (2)	98 (3)	98 (3)	98 (3)	98 (3)	1.0
Bicarbonate, mmol/L	41	11.1	28 (2)	29 (2)	28 (2)	28 (2)	28 (2)	0.286
Potassium, mmol/L	41	1.1	4 (0.3)	4 (0.2)	4 (0.3)	4 (0.3)	4 (0.3)	1.0
Sodium, mmol/L	41	1.0	138 (3)	138 (2)	138 (2)	138 (2)	138 (2)	1.0
Urea, mmol/L	41	18.9	4 (2)	4 (2)	4 (2.2)	4 (2)	4 (2)	1.0
Creatinine, µmol/L	41	6.3	71 (22.1)	71 (26.6)	71 (22.1)	71 (17.7)	71 (26.6)	1.0
Creatinine estimated glomerular filtration rate, mL/min/1.73m^2^	41	/	94 (16)	95 (17)	95 (16)	95 (16)	96 (16)	1.0
Calcium, mmol/L	41	5.0	2.32 (0.14)	2.31 (0.11)	2.32 (0.12)	2.32 (0.11)	2.31 (0.13)	1.0
Total protein, g/L	41	4.6	73 (6)	74 (6)	73 (7)	74 (5)	73 (6)	1.0
Glucose, mmol/L	41	4.8	94 (11)	93 (12)	94 (10)	93 (11)	93 (9)	1.0
C-reactive protein, mg/L	24 (17)	5.5	2 (3.25)	2 (3.25)	2 (3.25)	2 (3.25)	2 (3.25)	1.0
High sensitive C-reactive protein, mg/L	41	8.7	0.9 (1.4)	0.9 (1.5)	0.9 (1.4)	0.9 (1.6)	0.9 (1.5)	1.0
Uric acid, mmol/L	41	4.9	0.29 (0.12)	0.29 (0.12)	0.29 (0.11)	0.29 (0.11)	0.29 (0.11)	1.0
Phosphorus, mmol/L	41	6.5	0.97 (0.22)	0.97 (0.24)	0.96 (0.22)	0.98 (0.22)	0.97 (0.21)	1.0
Magnesium, mmol/L	41	5.5	0.82 (0.07)	0.82 (0.07)	0.82 (0.07)	0.83 (0.06)	0.82 (0.06)	1.0
Bilirubin total, µmol/L	41	4.0	8.6 (5.1)	8.6 (5.1)	8.6 (6.8)	8.6 (5.1)	8.6 (5.1)	1.0
Bilirubin direct, µmol/L	41	5.8	3.4 (2.4)	3.4 (0.7)	3.4 (0.7)	3.4 (0.9)	3.4 (0.9)	1.0
Cholesterol, mmol/L	41	5.8	5 (1.1)	5 (1.1)	5 (1.2)	4.9 (1.1)	5 (1)	1.0
Triglycerides, mmol/L	41	4.8	1 (0.6)	1 (0.6)	1.1 (0.6)	1 (0.6)	1.1 (0.6)	1.0
HDL-Cholesterol, mmol/L	41	6.2	1.8 (0.6)	1.8 (0.6)	1.8 (0.6)	1.8 (0.6)	1.9 (0.6)	1.0
Apo-Lipoprotein B, mg/L	41	3.9	940 (340)	940 (320)	950 (310)	930 (320)	940 (310)	1.0
Lipoprotein (a), nmol/L	32 (9)	4.0	27.8 (90)	28.8 (91.4)	28.2 (88.9)	27.8 (90.1)	28.4 (91.7)	1.0
Aspartate-transaminase, U/L	41	5.6	21 (8)	20 (9)	22 (8)	22 (9)	22 (7)	1.0
Alanine-transaminase, U/L	41	9.1	21 (14)	21 (11)	21 (12)	21 (14)	20 (13)	1.0
Lactate-dehydrogenase, U/L	41	1.6	164 (18)	158 (22)	170 (21)	171 (20)	157 (17)	**< 0.001**
Alkaline-phosphatase, U/L	41	6.7	57 (19)	56 (14)	56 (17)	55 (19)	57 (18)	1.0
γ-Glutamyl-transferase, U/L	41	2.7	15 (12)	14 (13)	14 (14)	14 (13)	14 (14)	1.0
Cholinesterase, U/L	41	2.2	74 (27)	73 (27)	74 (27)	74 (27)	73 (29)	1.0
Glutamate- dehydrogenase, U/L	40 (1)	4.8	2.3 (1.8)	2.6 (2.3)	2.5 (2.0)	2.6 (1.8)	2.5 (1.6)	1.0
Amylase, U/L	41	4.3	63 (21)	65 (21)	64 (21)	63 (21)	65 (20)	1.0
Lipase, U/L	41	9.7	32 (18)	33 (17)	33 (16)	33 (17)	32 (17)	1.0
Creatin-kinase, U/L	41	2.1	101 (90)	98 (92)	98 (92)	99 (92)	101 (94)	1.0
**Parameter, unit**	N*	**CV (%)^†^**	**Centrifugation conditions per tube type**					**P**
			**2000xg/10min**	**2000xg/10min**	**3000xg/7min**	**3000xg/5min**	**3000xg/5min**	
			**LiHepGel**	**LiHepBar**	**LiHepGel**	**LiHepGel**	**LiHepBar**	
Iron, µmol/L	41	2.4	17.2 (8.4)	17.4 (8.6)	17.2 (8.6)	17.2 (8.2)	17.4 (8.6)	1.0
Transferrin, µmol/L	41	2.0	32.1 (6.2)	32.5 (6)	32.6 (6.3)	32.1 (6.3)	32.3 (6.8)	1.0
Soluble transferrin receptor, nmol/L	41	2.9	34.9 (14.4)	34.1 (14)	34.1 (17.1)	34.6 (13.9)	34.3 (15.7)	1.0
Ferritin, µg/L	41	12.2	80 (120)	80 (127)	80 (124)	81 (126)	79 (126)	1.0
Fructosamine, µmol/L	41	12.7	256 (41)	261 (38)	256 (44)	258 (37)	264 (39)	1.0
Free haemoglobin plasma, g/L	41	/	0.06 (0.04)	0.05 (0.02)	0.07 (0.04)	0.07 (0.02)	0.04 (0.03)	**< 0.001**
Icterus index plasma, index	41	/	1 (0)	1 (0)	1 (0)	1 (0)	1 (0)	1.0
Lipemia index plasma, index	41	/	10 (6)	8 (6)	9 (5)	12 (6)	11 (5)	1.0
Creatin-kinase MB immunologic, µg/L	41	1.7	1.8 (1.2)	1.8 (1.2)	1.8 (1.1)	1.8 (1.2)	1.8 (1.2)	1.0
Myoglobin, µg/L	41	8.3	45 (14)	45 (15)	45 (15)	45 (15)	45 (14)	1.0
N-terminal pro brain natriuretic peptide, pmol/L	40 (1)	2.1	4.4 (4.7)	4.4 (4.4)	4.4 (4.3)	4.3 (4.6)	4.4 (4.2)	1.0
Thyroid stimulating hormone, mU/L	41	5.3	1.96 (1.17)	1.91 (1.15)	1.97 (1.12)	1.92 (1.2)	1.95 (1.13)	1.0
Free Triiodothyronine, pmol/L	41	6.4	4.7 (0.6)	4.6 (0.6)	4.6 (0.7)	4.6 (0.7)	4.6 (0.6)	1.0
Albumin, g/L	41	5.1	46 (3)	47 (3)	46 (3)	46 (3)	46 (4)	1.0
Complement C3, g/L	41	7.4	1.1 (0.2)	1.1 (0.2)	1.1 (0.2)	1.1 (0.2)	1.1 (0.2)	1.0
Complement C4, g/L	41	6.9	0.25 (0.12)	0.25 (0.12)	0.26 (0.11)	0.24 (0.13)	0.25 (0.11)	1.0
Alpha 1 – antitrypsin, µmol/L	41	4.9	24 (5)	24 (5)	24 (5)	24 (5)	24 (5)	1.0
Haptoglobin, g/L	41	6.2%	1.11 (0.66)	1.13 (0.66)	1.1 (0.66)	1.14 (0.69)	1.15 (0.65)	1.0
Ceruloplasmin, µmol/L	41	4.3%	1.5 (0.3)	1.5 (0.3)	1.5 (0.3)	1.5 (0.3)	1.5 (0.3)	1.0
Interleukin 6, pg/mL	41	1.9%	3.8 (1.5)	3.6 (1.3)	3.5 (1.3)	3.6 (1.3)	3.6 (1.6)	1.0
Data are presented as median and interquartile range. P < 0.05 was considered statistically significant. *Number of individual samples used for calculation. In those parameters where this value is less than 41, some of the samples had measured values below the measuring range of the respective assay. The number of excluded samples, where applicable, are shown in parentheses. ^†^All assays were performed on a COBAS 8000 instrument (Roche Diagnostics) using only proprietary reagents. CVs of day-to-day QC control values of the respective parameter. LiHepGel – Lithium Heparin plasma from tubes with gel separator. LiHepBar – Lithium Heparin plasma from tubes with mechanical separator. CV – coefficient of variation.								

Twice daily quality controls (QC) controls within the target range and Westgard rules (1_3s_, 1_2s_, 2_2s_, R_4s_ and 2of3_2s_) were a premise for analyses of the samples. Control samples from two different manufacturers were used (Roche Diagnostics, Basel, Switzerland and Bio-Rad Laboratories, Hercules, USA). Coefficients of variations (CV) for QC of all analytes tested are shown in Table 1 and Table 2. For calculation of these CVs we used data from the current lot of the low level control of respective parameters. If this lot was in use for less than two weeks, we additionally used the data of the previous lot. Haemolysis index (HI) measurements are referred to as free haemoglobin (fHb), since respective measurements from this instrument are directly correlated with an HI of 1 being equal to a fHb value of 0.01 g/L (*22*, *23*). If test results were below the measurement range, all according values of the other centrifugation settings from this specific sample were not considered for further analysis. If results from more than 50% of samples had to be excluded, the respective analyte was eliminated from statistical analyses.

**Table 2:** Comparison of laboratory values measured in serum for different centrifugation conditions

**Parameter**	N*	**CV (%)^†^**	**Centrifugation conditions**			**P**
**2000xg/10min**	**3000xg/7min**	**3000xg/5min**
Free haemoglobin, g/L	41	/	0.05 (0.03)	0.07 (0.04)	0.07 (0.03)	**0.001**
Icterus index, index	41	/	1 (0)	1 (0)	1 (0)	0.99
Lipemia index serum, index	41	/	5 (4)	6 (5)	6 (5)	0.99
Anti-Müllerian hormone, pmol/L	37 (4)	4.4	25.7 (23.5)	25.7 (23.5)	25.7 (23.5)	0.99
Prealbumin, µmol/L	41	5.7	4.8 (1.4)	4.9 (1.1)	4.8 (1.5)	0.99
Carcinoembryonic antigen, U/L	38 (3)	10.2	23.7 (16.1)	23.7 (14.8)	23.7 (14.8)	0.99
Alpha Fetoprotein, U/L	38 (3)	7.4	19.9 (13.1)	19.9 (14.5)	19.9 (12.9)	0.99
Cytokeratin fragment 21-1, µg/L	41	6.1	1.2 (0.7)	1.2 (0.8)	1.2 (0.7)	0.99
Cancer-Antigen 125, U/L	41	5.7	120 (60)	120 (80)	120 (70)	0.99
Cancer-Antigen19-9, U/L	41	6.2	70 (150)	70 (150)	60 (150)	0.99
Cancer-Antigen 15-3, U/L	41	6.4	170 (110)	170 (100)	170 (110)	0.99
S100 protein, µg/L	41	7.2	0.05 (0.02)	0.05 (0.02)	0.05 (0.02)	0.99
Neuron specific enolase, µg/L	41	6.2	11 (3)	12 (3)	12 (3)	0.71
Cancer-Antigen 72-4, U/L	41	6.5	13 (37)	13 (36)	13 (37)	0.99
Cystatin C, nmol/L	41	2.9	62.9 (11.2)	62.9 (12)	62.9 (12.7)	0.99
Cystatin C estimated glomerular filtration rate, mL/min/1.73m^2^	41	/	97 (19)	99 (19)	98 (20)	0.99
Soluble fms-like tyrosine kinase-1, pg/mL	41	16.0	89.4 (11.7)	89.9 (11)	88.8 (13.1)	0.99
Placental growth factor, pg/mL	41	16.8	14.89 (4.5)	14.64 (4.2)	14.95 (4.2)	0.99
sFlt1/PlGF Ratio, ratio	41	/	6.29 (1.6)	6.4 (1.6)	6.4 (1.6)	0.99
Data are presented as median and interquartile range. P < 0.05 was considered statistically significant. *Amount of individual samples used for calculation. In those parameters where this value is less than 41, some of the samples had measured values below the measuring range of the respective assay. The number of excluded samples, where applicable, are shown in parentheses. ^†^All assays were performed on a COBAS 8000 instrument (Roche Diagnostics) using only proprietary reagents. CVs of day-to-day QC control values of the respective parameter. CV – coefficient of variation.						

### Statistical analysis

Linear mixed effect modelling with Benjamini-Hochberg multiple testing correction was performed for different centrifugation conditions (fixed effects for centrifugation condition and sex, random effect for replicate). As a *post-hoc* test for our linear mixed effects modelling we calculated pairwise differences of least squares means for the fixed effects of interest. For the parameters with statistically significant differences, agreement analyses using the total allowable error (derived from the Ricos criteria on biological variation) as cut-off for the total deviation index (TDI) and targeting at a coverage probability (CP) of 80% were performed as suggested by Barnhart *et al.* (*24*, *25*). Normality testing was done using the Anderson Darling test. All statistical analyses were performed with SPSS 23.0 (SPSS Inc. Chicago, USA), R v.3.4.1 (The R Foundation, Vienna, Austria) and python scripts (Python Software Foundation, Python Language Reference, version 3.6). *A-priori* sample size calculation was performed using G-Power V3.1.9.2 (Heinrich-Heine-University, Düsseldorf, Germany) yielding a statistical power of 0.8 (*26*).

## Results

Measurements of C-reactive protein (CRP), lipoprotein a (Lp(a)), N-terminal pro brain natriuretic peptide (NT-proBNP), glutamate-dehydrogenase (GLDH), anti-streptolysin O (ASL), anti-Müller hormone (AMH), carcinoembryonic antigen (CEA) and alpha fetoprotein (AFP) yielded values below the measuring range in some samples. Subsequently, respective samples were not used for further calculations. Since samples were collected in a healthy group of volunteers, high sensitive Troponin T (hsTnT), pregnancy-associated plasma protein A (PAPP-A), ß human chorionic gonadotropin (ßHCG) and rheumatoid factor (RF) were below the measuring range in over 50% of study participants samples and were therefore excluded from calculations. For all other parameters, values within the measuring range could be obtained in samples of all 41 subjects (Table 1 for analytes measured in plasma and Table 2 for analytes measured in serum). As some of the parameter values were normally distributed while others were not, we chose to depicture all values as median and interquartile range (IQR).

**Figure 2:**
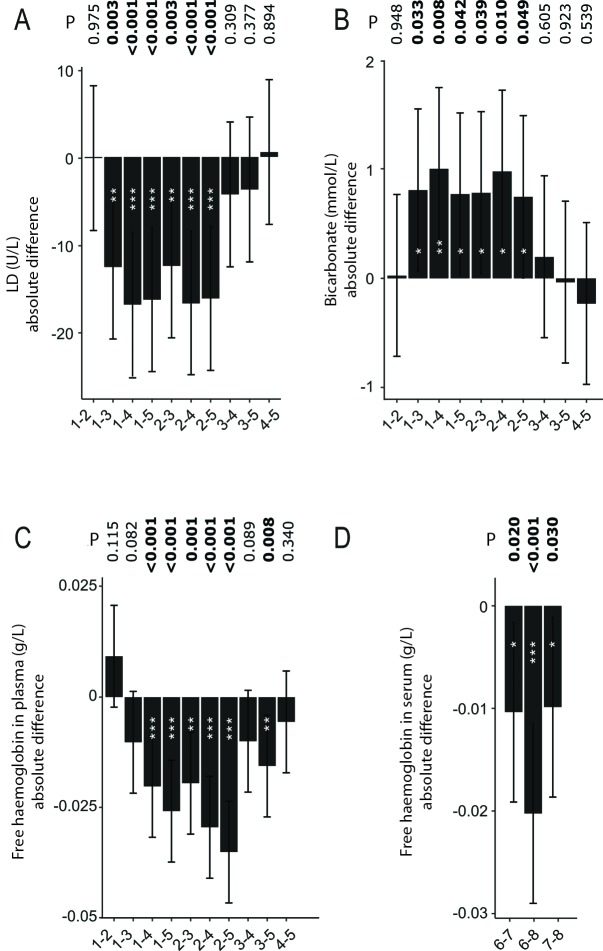
Comparison of absolute results of lactate-dehydrogenase (A), bicarbonate (B), free haemoglobin in plasma (C) and serum (D) between centrifugation settings and tube types. Scales on the y-axis represent differences in absolute values of the respective analyte. IDs on the x-axis represent the different centrifugations settings: 1 – LiHepBar 2000xg/10min; 2 – LiHepBar 3000xg/5min; 3 – LiHepGel 2000/10min; 4 – LiHepGel 3000xg/5min; 5 – LiHepGel 3000xg/7min; 6 – Serum 2000xg/10min; 7 – Serum 3000xg/5min; 8 – Serum 3000xg/7min. *P < 0.05; **P < 0.01; ***P < 0.001. P-values < 0.05 were considered statistcally significant.

Almost no parameters showed any significant differences after overall multiple testing corrections. Only lactate-dehydrogenase (LD) and measurements of fHb in serum (fHb_S) and plasma (fHb_P) showed respective overall multiple testing-corrected P-values of less than 0.05 (Table 1 and Table 2). A more detailed analysis of LD results revealed that the observed differences in measurements originated from the tube types (LiHepGel and LiHepBar) irrespective of the centrifugation conditions (Figure 2A). Bicarbonate (BIC) measurements, which were non-significant after overall multiple-testing correction (P = 0.286), also showed slight yet significant differences when comparing tube types (Figure 2B). Measurement of fHb_P and fHb_S differed between tube types and also between centrifugation settings (Figure 2C and 2D). When testing for clinical significance of these differences using the agreement analysis and a cut-off of 11.4% (total allowable error according to Ricos criteria) for LD, the coverage probability to get the same result in LiHepBar *vs* LiHepGel tubes was ~ 60% and ~ 50% for centrifugation at 2000xg for 10 minutes and 3000xg for 5 minutes, respectively. This means that in both cases we did not get an agreement between the measurements (the probability of values differing between these tubes by more than 11.4% at the same centrifugation setting is greater than 40%) and differences in LD measurements between LiHepGel and LiHepBar tubes are most probably to be considered clinically significant.

## Discussion

In our study we could show that apart from fHb, none of the 77 analytes investigated differed significantly between centrifugation settings (Table 1, Table 2 and Figure 2). Looking more closely into these fHb differences we found that the higher centrifugation speed of 3000xg is associated with higher fHb concentrations (Figure 2C comparison 3-5 and figure 2D comparisons 6-8), as already mentioned by Lippi *et al.* (*1*). However, when reducing the time of centrifugation to 5 minutes also at 3000xg, these significances were diminished (Figure 2D comparisons 6-7) or even vanished (Figure 2C comparison 3-4). As these differences in fHb values between centrifugation settings were rather small and did not seem to have any influence on the tested clinical chemistry analytes, our findings suggest that respective samples for these analyses can be centrifuged at 3000xg for 7 or 5 minutes without a significant alteration of test results. However, a raise in fHb even on a low level might have impact on analytical outcome if this increase contributes to a final fHb concentration above an analyte specific haemolysis cut-off, subsequently leading to a deletion of the respective result.

In order to compare our results to other studies, we scanned the literature for evidence based recommendations and found these to be very scarce regarding clinical chemical analytes, finding only expert opinion suggestions ranging from 10 to 20 minutes and 1500 to 3000xg at 15 to 25 °C, respectively (*6*-*10*). For other parameters such as coagulation analyses however, several studies have been undertaken aiming at reducing centrifugation time at higher g-forces (*3*, *4*, *17*, *18*, *27*, *28*). Considering the potential analytical consequences, it seems odd that no real standardization has been achieved for this step while the analytical processes are constantly improved and accurately controlled. However, there was one investigation carried out by Møller *et al.* in which nine selected parameters as well as haemolysis, lipemia and icterus index were measured in serum and lithium heparin sample pairs from 40 patients. Each of these sample pairs were centrifuged at 2200xg for 10 minutes and at 3000xg for 5 minutes (*19*). Similar to our findings, the authors could show a good correlation of analyses. However, conflicting with our data, they found LD measurements to increase by 6.3% on average at higher centrifugation speed. Maybe the difference in study population contributed to this fact, as the authors collected blood from hospitalized patients whereas we drew blood from healthy volunteers with lower LD ranges. Also the analytical platform differs as we performed analyses on a Roche COBAS system and Møller *et al.* used an Abbott Architect system, in which haemolysis is reported in levels rather than as quantitative value (*29*). A statistical evaluation of these levels therefore may differ from ours.

In combination with findings of Sedille-Mostafaie *et al.* demonstrating that blood for coagulation testing can be centrifuged at 3000xg for 7 minutes without altering measurement values in a broad variety of haemolysis analytes, samples for clinical chemistry and coagulation analyses could eventually be centrifuged simultaneously (*16*). As many laboratories process samples using automated processes, combining pre-analytical and analytical procedures, this could help reducing intra-laboratory TAT as well as the number of centrifuges needed.

Besides these findings we also evaluated the newly introduced BD Barricor tubes (LiHepBar), which use a mechanical element to separate plasma from cellular components during centrifugation. Comparing measurements between centrifugation settings in these tubes, we did not find a significant difference in any of the tested analytes, including fHb. However, comparing analysis results between LiHepBar and the commonly used LiHepGel tubes, we found differences in fHb, LDH and BIC measurements, performed in plasma retrieved from identical centrifugation settings (Figure 2 A-C). Since the mechanical separator within the LiHepBar tubes allows cellular blood components to pass during the entire centrifugation process and gel separators in the respective tubes occlude this passage already at an early stage of centrifugation, we hypothesized that differences in residual cell count may be the reason for the disagreement in fHb and LDH values between these two tubes. In a small explorative experiment we could strengthen our assumption that LDH levels may be increased upon cellular release (data not shown), however, a separate study would be needed to conclusively prove this causal relationship. Nevertheless, according to our data, separate LDH reference values for LiHepBar tubes appear to be necessary.

For the minor, yet significant BIC differences of about 1 mmol/L between these tubes we could not find a definite reason. Although respective samples in this study were treated identically, we assume that the exposure to air in these tubes or differences in proceeding cellular metabolism might be contributing to this fact. However, as we could not find similar results in current literature, we were not able to confirm or compare our findings.

As limitation to this study, we want to mention the fact that analyses, which are only increased in certain medical conditions (hsTnT, RF, ßHCG, PAPP-A) could not be investigated properly, since only healthy volunteers were tested. Subsequently, we could also not cover specific medical conditions possibly affecting sample stability in different centrifugation conditions.

In conclusion, in this study, we provide evidence that samples for the measurement of 77 clinical chemistry analytes may be centrifuged at 3000xg for 7 or 5 minutes without any impact on test results compared to centrifugation at 2000xg for 10 minutes, yielding a benefit of up to 5 minutes in intra-laboratory TAT. As residual leucocyte count, fHb and subsequently lactate dehydrogenase results are lower in the BD Barricor tubes, a new LDH reference range might be necessary when using these tubes.
